# Outside the Safe Operating Space of a New Planetary Boundary for Per- and Polyfluoroalkyl Substances (PFAS)

**DOI:** 10.1021/acs.est.2c02765

**Published:** 2022-08-02

**Authors:** Ian T. Cousins, Jana H. Johansson, Matthew E. Salter, Bo Sha, Martin Scheringer

**Affiliations:** † Department of Environmental Science, Stockholm University, SE-10691 Stockholm, Sweden; ‡ Institute of Biogeochemistry and Pollutant Dynamics, ETH Zürich, 8092 Zürich, Switzerland; § RECETOX, Masaryk University, 625 00 Brno, Czech Republic

**Keywords:** PFAS, planetary boundary, chemical pollution, environmental exposure

## Abstract

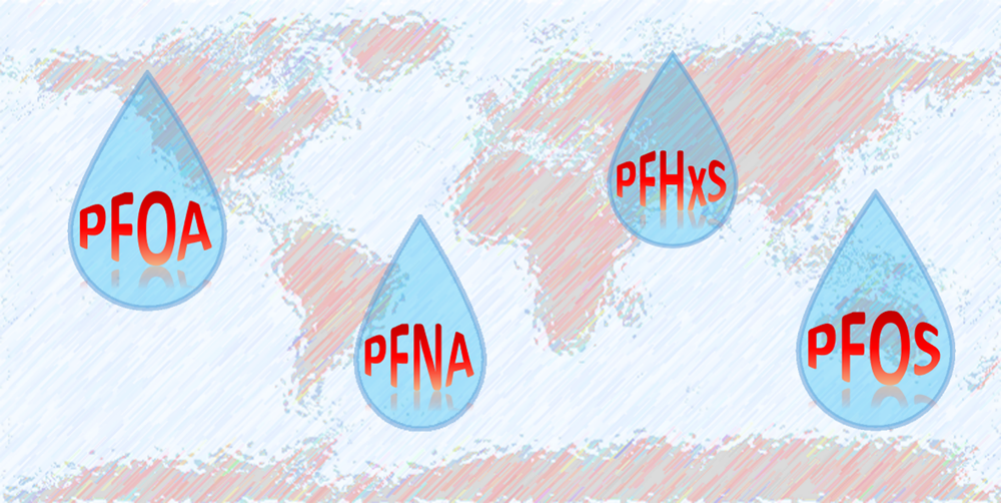

It is hypothesized that environmental contamination by per- and polyfluoroalkyl substances (PFAS) defines a separate planetary boundary and that this boundary has been exceeded. This hypothesis is tested by comparing the levels of four selected perfluoroalkyl acids (PFAAs) (i.e., perfluorooctanesulfonic acid (PFOS), perfluorooctanoic acid (PFOA), perfluorohexanesulfonic acid (PFHxS), and perfluorononanoic acid (PFNA)) in various global environmental media (i.e., rainwater, soils, and surface waters) with recently proposed guideline levels. On the basis of the four PFAAs considered, it is concluded that (1) levels of PFOA and PFOS in rainwater often greatly exceed US Environmental Protection Agency (EPA) Lifetime Drinking Water Health Advisory levels and the sum of the aforementioned four PFAAs (Σ4 PFAS) in rainwater is often above Danish drinking water limit values also based on Σ4 PFAS; (2) levels of PFOS in rainwater are often above Environmental Quality Standard for Inland European Union Surface Water; and (3) atmospheric deposition also leads to global soils being ubiquitously contaminated and to be often above proposed Dutch guideline values. It is, therefore, concluded that the global spread of these four PFAAs in the atmosphere has led to the planetary boundary for chemical pollution being exceeded. Levels of PFAAs in atmospheric deposition are especially poorly reversible because of the high persistence of PFAAs and their ability to continuously cycle in the hydrosphere, including on sea spray aerosols emitted from the oceans. Because of the poor reversibility of environmental exposure to PFAS and their associated effects, it is vitally important that PFAS uses and emissions are rapidly restricted.

## Abstract 2

A planetary boundary has been exceeded due to PFAS levels in environmental media being ubiquitously above guideline levels.

## Introduction

A recent review article in *Science*(1) highlighted the global threat posed by plastic pollution. These concerns were based on the high environmental persistence of plastics, the related “poor reversibility” and a range of potential effects. Other researchers, including ourselves,^2,3^ have pointed out similar concerns related to highly persistent nonpolymeric substances, but these concerns are not equally obvious to the public compared to the concerns with plastics. The relatively high public concern regarding plastics is possibly driven by the visibility of plastic waste compared to nonpolymeric substances.^4^ Clearly, both plastic pollution and pollution by highly persistent nonpolymeric substances lead to similar global problems. Persistence is generally seen as a less immediate hazardous property than toxicity, but it actually is the key factor that lets pollution problems spiral out of control.^2^ This is because persistence enables chemicals to spread out over large distances, causes long-term, even life-long exposure, and leads to higher and higher levels in the environment as long as emissions continue. These increasing levels will with high probability sooner or later lead to adverse effects. Importantly, microplastic is under consideration for restriction in the EU because of the extreme persistence of plastics and the irreversibility of the exposure caused by plastic particles in the environment.^5^

Recently a group of scientists flagged the concerns regarding the inability of scientific analyses to keep pace with the amount of chemicals produced and released into the environment,^6^ which limits the ability to discover new environmental threats in time. Others have similarly pointed out the need for precautionary chemicals managements; a notable example is the report, “Late Lessons from Early Warnings”,^7^ where many historical examples of global contamination problems are provided, often associated with persistent chemicals.

A well-known class of pollutants, the per- and polyfluoroalkyl substances (PFAS), have also recently featured in a review in *Science.*(8) The vast majority of PFAS are highly persistent (based on the EU REACH definition whereby a substance is persistent if it is persistent itself or has persistent degradation products^9^), and this has been seen as basis for managing them as a chemical class.^3^ While the review article in *Science* pointed out the ubiquity and high persistence of PFAS, it did not point out the current widespread and poorly reversible risks associated even with low-level PFAS exposures. It is hypothesized here that due to the global spread of PFAS, the irreversibility of exposure to PFAS, and the associated biological effects, a new planetary boundary for PFAS has been exceeded.

Unfortunately, although there are many thousands of substances defined as PFAS in use (PFAS include any substance with at least one −CF_2_– or −CF_3_ moiety in its structure^10^), the current understanding of biological impacts is based primarily on studies of four perfluoroalkyl acids (PFAAs), namely, perfluorooctanesulfonic acid (PFOS), perfluorooctanoic acid (PFOA), perfluorohexanesulfonic acid (PFHxS), and perfluorononanoic acid (PFNA). Whereas all PFAS can be grouped into a class on the basis of their high persistence,^3^ it is not possible to group many of them according to biological risk because of a paucity of data on exposure and effects for most PFAS.^11^ Therefore, because of data gaps, the analysis presented here is based only on the four PFAAs mentioned above. In the following, we provide four pieces of evidence to support the claim that, even considering only these four PFAAs, the new planetary boundary for PFAS has been exceeded.

In the planetary boundary concept, an attempt is made to estimate the boundaries for “a safe operating space for humanity with respect to the functioning of the Earth System”.^12,60^ Chemical pollution was one of the original nine anthropogenic impacts for which planetary boundaries were postulated because it can influence Earth System functioning: “(i) through a global, ubiquitous impact on the physiological development and demography of humans and other organisms with ultimate impacts on ecosystem functioning and structure and (ii) by acting as a slow variable that affects other planetary boundaries.”^12,60^ The “chemical pollution” boundary was renamed as the “novel entities” (NEs) boundary by Steffen et al.,^56^ where NEs are defined as “new substances, new forms of existing substances and modified life forms”, including “chemicals and other new types of engineered materials or organisms not previously known to the Earth system as well as naturally occurring elements (for example, heavy metals) mobilized by anthropogenic activities”. Several groups of scientists^6,57,58^ have pointed out the challenges in quantifying the planetary boundary for NEs, and recently it was proposed to instead use various control variables to determine if the boundary is exceeded.^6^ It is, in our opinion, an insurmountable task to quantify the boundary for all NEs because (1) there are critical data gaps for a large proportion of existing NEs, (2) NEs of various types and mixtures of NEs are continuously being generated and released to the environment, and (3) there are multiple possible effects (not only toxic effects) that individual NEs or groups/mixtures of NEs can cause. Several of the existing planetary boundaries are related to the release of NEs. For example, the boundaries for “stratospheric ozone depletion” and “climate change” address the release of ozone depleting substances and gases with global warming potential, respectively. Therefore, rather than being a single planetary boundary, the boundary for NEs can be thought of as a placeholder for multiple planetary boundaries for NEs that may emerge. It is argued here that PFAS define a new planetary boundary for NEs.

We argue that if drinking water health advisories and other guidelines designed to protect human health are exceeded due to the global environmental spread of PFAS, then there is a real danger of global health effects (e.g., affecting human physiology) occurring and that it can be argued that the planetary boundary for PFAS is exceeded. We do not deem it necessary to demonstrate the prevalence of global human health effects due to PFAS exposure to prove our hypothesis, and we hope that such widespread effects in the human population are never observed.

## The US EPA Lifetime Drinking Water Health Advisories for PFOS and PFOA Are Often Lower than Their Respective Levels in Rainwater and the Danish Drinking Water Limit Value for Σ4 PFAS Is Also Often Lower than the Level of Σ4 PFAS in Rainwater

In June 2022, the US Environmental Protection agency (EPA) announced the release of health advisories for four PFAS, including interim updated nonregulatory lifetime drinking water health advisories for PFOA and PFOS of 4 pg/L and 20 pg/L, respectively.^13^ The US EPA health advisories identify the concentration of chemicals in drinking water at or below which adverse health effects are not anticipated to occur and, in divergence with previous advisories, are based on human epidemiology studies in populations exposed to these chemicals. The most sensitive noncancer effect and the basis for the risk assessment behind the interim updated health advisories for PFOA and PFOS is suppression of vaccine response (decreased serum antibody concentrations) in children. The US EPA’s previous nonregulatory lifetime drinking water health advisories were 70 ng/L for the sum of concentrations of PFOS and PFOA. In 2020, the European Food Safety Authority (EFSA) published their Opinion on the risks to human health arising from the presence of PFAS in food^14^ and proposed a group tolerable weekly intake (TWI) of 4.4 ng/kg body weight for the sum of PFOA, PFNA, PFHxS, and PFOS. On the basis of the available studies in animals and humans, effects on the immune system were considered the most critical for the basis of the risk assessment.^14^ In June 2021, on the basis of the TWI in the EFSA Opinion, the Danish Environmental Protection Agency tightened their drinking water limit values and announced that drinking water must not contain more than 2 ng/L of Σ4 PFAAs.^15^

PFAS drinking water guidelines have progressively decreased over the last 22 years.^16^ For example, in the US the PFOA drinking water guideline for West Virginia was 150 000 ng/L,^16^ which is higher by a factor of 37.5 million than the recently announced US EPA drinking water lifetime advisory for PFOA of 4 pg/L. As a result of this decrease, international drinking water guidelines for PFAS are now close to, or even lower than, levels in precipitation. Humans residing in industrialized areas of the world do not often drink rainwater in modern life, but it should nevertheless be a reasonable expectation that the environment is clean enough that rainwater and mountain stream water fed by precipitation is safe to drink. Furthermore, in some parts of the world, notably in some arid and tropical regions, rainwater remains an important source of drinking water.^59^

**Figure 1:**
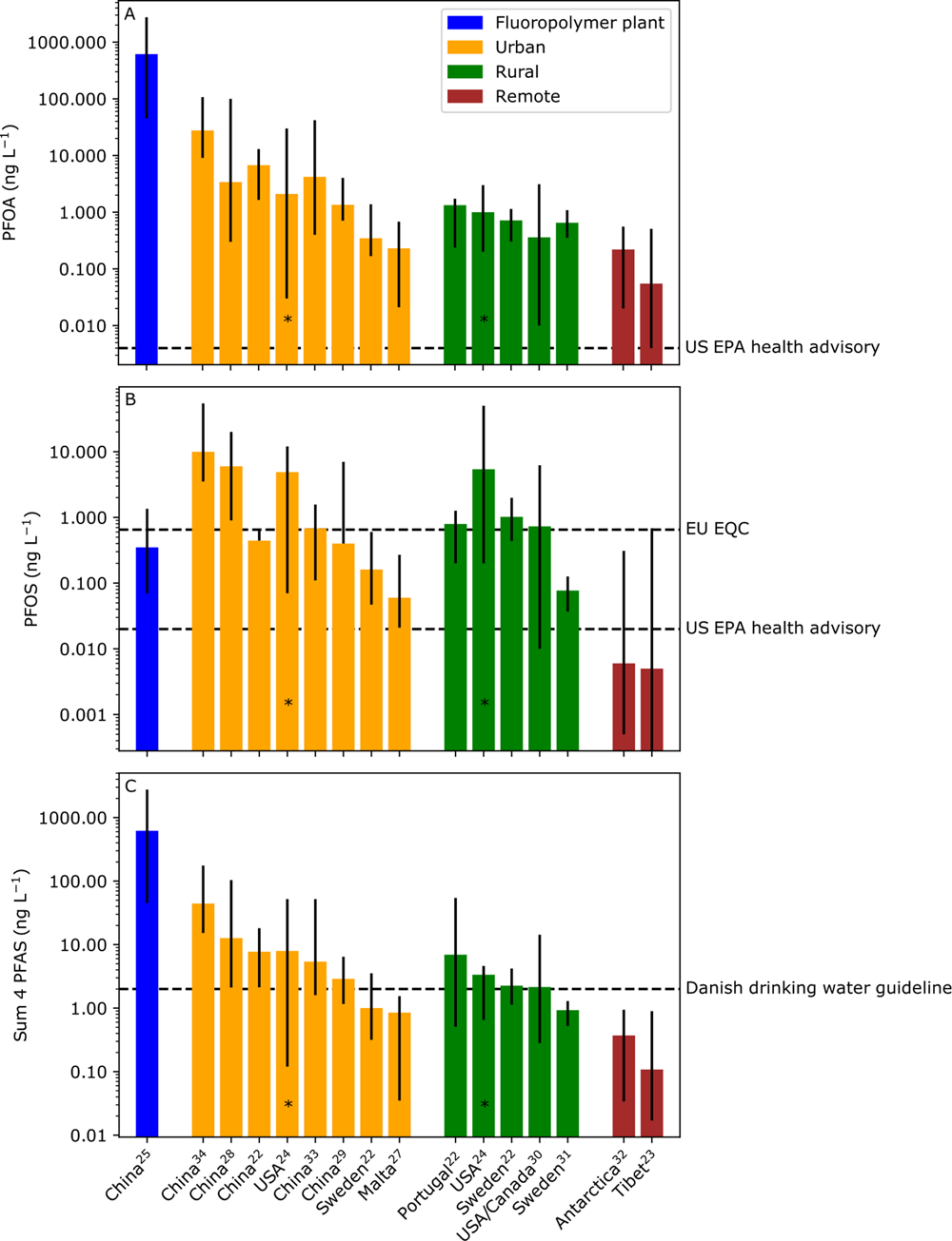
Levels of (A) PFOA, (B) PFOS, and (C) Σ4 PFAAs (PFOA + PFNA + PFHxS + PFOS) in wet deposition collected at various global locations from 2010 to the present. For one study,^19^ it was not possible to derive median values and thus mean values are provided (indicated by *). The dashed line in (A) shows the US EPA health advisory for PFOA (0.004 ng/L), the dashed lines in (B) show the EU EQC for PFOS (i.e., 0.65 ng/L) and the US EPA health advisory for PFOS (i.e., 0.020 ng/L), and the dashed line in (C) shows the Danish drinking water guideline for Σ4 PFAAs (i.e., 2 ng/L). Bars indicate median values, and the uncertainty bars indicate minimum and maximum values. Wet deposition measurements for Σ4 PFAAs are ordered from high to low (from left to right) and sorted into four categories (“Fluoropolymer plant” indicates that samples were taken close to a fluoropolymer manufacturing plant; “Urban” indicates that samples were taken in cities or urbanized regions; “Rural” indicates that samples were taken in rural less-populated locations, and “Remote” indicates that samples were taken in regions with very low or nonexistent human populations). Some studies sampled wet deposition in multiple locations within one of the four categories, and thus data from these individual locations are grouped together in several bars. The raw data and a description of data treatment for figure preparation are provided in the Supporting Information.

In Figure 1, the levels of PFAS in precipitation are reviewed and compared to drinking water advisories for Denmark and the US EPA, which are the most stringent advisories known globally. The criteria for including/excluding studies for the selection shown in Figure 1 are (1) only studies which have precipitation samples are considered, (2) sampling and analysis was carried out after 2010, and (3) raw data or descriptive statistics (range and median or mean concentration) of the data were provided. Only data from 2010 or later were included because (1) these data are more recent and further from the 2000–2002 3M phase-out of long-chain PFAS chemistries and (2) there were large analytical improvements throughout the early 2000s as evidenced by the improvement in the fourth international interlaboratory study of 2011 compared to the three international interlaboratory studies conducted between 2004 and 2009.^17^ Four precipitation studies were excluded because although the studies were published after 2010, the analysis was performed prior to 2010 (see Supporting Information).

In Figure 1A, the levels of PFOA in rainwater greatly exceed the US EPA drinking water health advisory for PFOA, even in remote areas (the lowest value for PFOA is for the Tibetan Plateau with a median of 55 pg/L,^18^ which is approximately 14 times higher than the advisory). In Figure 1B, the levels of PFOS in rainwater are shown to often exceed the US EPA drinking water health advisory for PFOS, except for two studies conducted in remote regions (in Tibet and Antarctica). In Figure 1C, the levels of Σ4 PFAAs in precipitation are reviewed,^18−29^ and it is shown that, in populated regions (defined as “urban” and “rural” in Figure 1), the levels would often exceed the Danish limit values for drinking water. In remote regions, with low human populations, the Σ4 PFAAs in rainwater also often exceeds the Danish drinking water limit value (Figure 1C). In Sweden, a national mapping of PFAS in municipal raw and drinking waters was undertaken in 2021.^30^ About 49% of drinking waters in Sweden were found to contain ΣPFAS > 5 ng/L, and it was shown that the 4 PFAAs that are included in EFSA’s risk assessment contributed a large fraction of the total PFAS measured. For comparison with the US, it was recently estimated^61^ that at a concentration of 5 ng/L for combined PFOA + PFOS, 21−123 million people or 7−41% of the US population may have drinking water at or above that level, and at a concentration of 2.5 ng/L, it was estimated to be 76−205 million people or 25−68% of the population. The Swedish drinking water guideline for mitigation action (90 ng/L for Σ11 PFAS)^31^ was previously based on the 2008 EFSA Scientific Opinion on PFOS and PFOA^32^ and was recently reduced to 4 ng/L Σ4 PFAAs,^31^ in light of the 2020 EFSA Opinion on PFAS.^14^

The US EPA health advisories seem not to be practically reachable without investment of huge cleanup costs in drinking water treatment plants given that most drinking water sources on the planet will have PFAS levels above the advisory levels. The US EPA health advisories are nonregulatory but demonstrating compliance to these guidelines would be an analytical challenge because modern methods are typically not able to achieve detection limits for PFOA below 4 pg/L in drinking water. Modern research laboratories have detection limits as low as 80 pg/L for PFOA and 100 pg/L for PFOS, respectively, in drinking water^33^ and commercial laboratories tend to have much higher detection and quantification limits (e.g., Eurofins has quantification limits of about 1 ng/L for PFAS in water^34^). Achieving detection limits of <4 pg/L for PFOA in drinking water would be theoretically possible given that low pg/L levels have been previously measured in ocean water samples, even more than a decade ago.^35^ Achieving such a low detection limit in drinking water would probably require extraction of larger than typical sample volumes and/or injection of larger extract volumes on the instrument. For example, the published method^33^ that achieved 80 pg/L detection limits for PFOA was based on 10 mL samples and could be scaled to achieve <4 pg/L detection limits with larger sample volumes. It will also be important to have very low blank contamination because ultimately the blank levels and associated quality assurance will determine the detection limits that can be achieved.

## The European Union (EU) Environmental Quality Standard (EQS) for PFOS for Freshwaters Is Often Lower than Levels in Rainwater

In 2010, the National Institute for Public Health and the Environment (RIVM) in the Netherlands derived a risk-based maximum permissible concentration (MPC) for PFOS in freshwaters of 0.65 ng/L based on potential for secondary poisoning in humans due to fish consumption.^36^ The MPC is a guideline level and it is defined as “the level at which no harmful effects are expected, based on annual average concentrations”. In 2013, PFOS and its derivatives were included in Directive 2013/39/EU and thus considered “Priority Hazardous Substances” under the Water Framework Directive (WFD) (2000/60/EC). Environmental Quality Standards (EQC) were, then, set for PFOS and its derivatives for freshwaters, marine waters, and biota. The EU annual average environmental quality standard (AA-EQS) for PFOS in Inland EU Surface Water was set at 0.65 ng/L, following the same reasoning used previously by RIVM. It is known that concentrations of PFOS in freshwaters regularly exceed the EQS,^37,38^ but potentially of more concern is that the levels of PFOS in *rainwater* are equal to, or even exceed the EQS. As can be seen in Figure 1A), the levels of PFOS in rainwater in populated regions in the northern hemisphere in some cases exceed, or are close to, the EQC of 0.65 ng/L. Therefore, regardless of wastewater inputs to freshwaters, the EQC for PFOS will likely always be approached in populated regions, and often exceeded, as a result of the widespread presence of PFOS in atmospheric deposition.

Recently, authorities in the Stockholm metropolitan region have advised the public not to eat fish from lakes in the region.^39^ This was not based on exceedance of the 0.65 ng/L EQS for PFOS and associated secondary poisoning but rather on exceedance of a temporary action level for fish of 9.1 ng/g PFOS set by the Swedish Food Agency.^31^ The Swedish action level is considered temporary because it will be revised in the near future^31^ according to the 2020 EFSA Scientific Opinion on the risks to human health arising from the presence of PFAS in food.^14^ Given that the EU freshwater EQC is based on secondary consumption in humans because of fish consumption, there are grounds for revising the EQS based on the recent EFSA Opinion.^14^ Such a revision of the freshwater EQS would likely result in a further reduction in its level and in basing the EQS on the sum of PFOA, PFNA, PFHxS, and PFOS.

## The Dutch Guidelines for PFAS in Soils and Dredging Material Were Impossible to Apply Due to the Ubiquity of PFAS in Atmospheric Deposition

Recent guidelines set in July 2018 by the infrastructure ministry in the Netherlands stated that soil and dredging material should not contain concentrations of >0.1 μg/kg dry weight (dw) of either PFOS or PFOA.^40^ As the levels of PFAS in soils often exceeded these guideline values, 70% of building projects involving soil removal and filling with excavated material were halted in the Netherlands.^41^ Following builders’ protests, the Dutch government relaxed the guidelines.^42^ Only a few studies have reported levels of PFAS in soils that have no known local PFAS source nearby. For example, Rankin et al. reported median PFOS and PFOA concentrations of 0.47 and 0.12 μg/kg dw for global soils,^43^ whereas Sörengård et al. reported median PFOS and PFOA concentrations of 0.39 and 0.38 μg/kg dw in Swedish forest soils.^44^ These reported soil levels illustrate the impossibility of complying with the Dutch guidelines before they were revised upward. The background soil contamination with PFAS is again a result of the environmental ubiquity of PFAAs in atmospheric deposition. If soils are amended with sewage sludge or biosolids, which is a common practice in agriculture in many countries, then soil levels will be further elevated and PFAS can leach to contaminate surface water and groundwater, including drinking water sources. On the basis of concerns regarding PFAS soil contamination, the US State of Maine passed a bill banning the use of biosolids in land applications unless, in the unlikely case, they could be shown to be PFAS free.^45^

## The Cycling of PFAAs in the World’s Hydrosphere Means That Levels of PFAAs in Rainwater Will Be Practically Irreversible

Until recently, the common belief was that PFAAs would eventually wash off into the oceans where they would stay to be diluted over the time scale of decades.^46^ A recent study,^47^ however, has provided evidence that certain PFAS, notably the long-chain PFAAs, which include the 4 PFAAs included in EFSA’s TWI, can be significantly enriched on sea spray aerosols (SSA) and transported in the atmosphere back to shore where they will be deposited and contaminate freshwaters, drinking waters and surface soils.

This continual global cycling of PFAAs in the hydrosphere will lead to the continued exceedance of the above-mentioned guidelines. This finding is particularly worrying because (1) guideline values based on biological effects have continually decreased^16^ and may not yet have reached the bottom as more scientific evidence emerges, (2) guidelines are currently based on only a few of the substances in the large PFAS class,^10^ and (3) there is no evidence for the decline in environmental concentrations and thus environmentally derived exposures of PFAS.^48^

## Discussion

PFAS are a planetary boundary problem based on the criteria outlined by MacLeod et al.,^49^ namely, (1) the diffuse PFAS pollution is global in its scale, (2) the effects are only now being discovered after the pollutants are already globally spread, and (3) now that the effects have been discovered they are poorly reversible or irreversible. As with most chemicals in use,^6^ because of the lack of information, it is impossible to make a full assessment of the planetary boundary threat for the many thousands of PFAS in the class. Nevertheless, based on the four PFAAs considered here, it is concluded that in many areas inhabited by humans the planetary boundary for PFAS has been exceeded based on the levels in rainwater, surface water and soil, with all of these media being widely contaminated above recently proposed guideline levels. Although the global emissions of these 4 PFAAs have been reduced in recent years in most countries,^46,50^ these substances continue to remain in the environment due to their high persistence and will continually cycle in the hydrosphere.

The analysis presented here has purposefully referred to the most stringent PFAS guideline values on an international basis, which are not representative of international guideline values for PFAS. There is, for example, a large disagreement internationally, and even between individual states in the US,^16^ regarding drinking water guidelines for PFAS. The various guidelines were developed by different scientists at different time points and the risk assessments are often based on varying end points. A clear and disturbing temporal trend emerges, however, with more recent guidelines being several orders of magnitude lower than older guidelines.^16^ Guidelines in the US and Europe have been driven downward recently as a result of emerging evidence for the suppression of vaccine response in children.^51^ We make no attempt to determine which of the many guidelines (see compilation^52^) is based on the strongest empirical evidence on effects because such a judgment is outside of our expertise. The point that we want to make is that the most stringent risk-based health advisories are often well below environmental levels, and this should be of concern and a reason for taking stringent measures.

Although PFAS are globally present in all environmental media and locations, there are still some few areas of the planet where the environmental levels of PFAS remain relatively low. However, even in these remote and sparsely populated regions, such as Antarctica and the Tibetan plateau, the most stringent PFAS guidelines are exceeded (Figure 1). These areas cannot support large populations and are not available for settlements where major parts of the population could move. In most other areas, PFAS guideline values are exceeded and this implies potential public health impacts: higher incidences (notably in large populations, i.e., many cases) of PFAS-related effects, such as reduced immune response, but also high additional costs for healthcare and, where possible, remediation.^53^ Moreover, in many cases, PFAS-related impacts occur in combination with other environmental issues, such as water scarcity or pollution by other contaminants.

Finally, we conclude that PFAS define a new planetary boundary that has been exceeded, based on PFAS levels in environmental media being ubiquitously above guideline levels. Irrespective of whether or not one agrees with our conclusion that the planetary boundary for PFAS is exceeded, it is nevertheless highly problematic that everywhere on Earth where humans reside recently proposed health advisories cannot be achieved without large investment in advanced cleanup technology. Indeed, although PFOS and PFOA were phased out by one of the major manufacturers (3M) 20 years ago, it will take decades before levels in land-based water and precipitation approach low picogram per liter levels. Moreover, the problems associated with PFOS, PFOA, or Σ4 PFAAs are likely to be only the tip of the iceberg given that there are many thousands of PFAS in the class and the risks associated with most of them are unknown.^54^ In view of the impacts of humanity’s chemical footprint on planetary health, it is of great importance to avoid further escalation of the problem of large-scale and long-term environmental and human exposure to PFAS by rapidly restricting uses of PFAS wherever possible.^55^ Furthermore, as has been stated by ourselves^3^ and others^7^ before, society should not continually repeat the same mistakes with other persistent chemicals.

## Supporting Information Available

Raw data and references used to create Figure 1 (XLSX)
